# Primary school teachers’ misconceptions about Attention Deficit/Hyperactivity Disorder in Nekemte town, Oromia region, Western Ethiopia

**DOI:** 10.1186/s13104-019-4573-9

**Published:** 2019-08-20

**Authors:** Ashenafi Habte Woyessa, Thanasekaran Palanichamy Tharmalingadevar, Shivaleela P. Upashe, Dereje Chala Diriba

**Affiliations:** 1 Department of Emergency and Critical Care Nursing, Wollega University, P. O. Box: 395, Nekemte, Oromia Ethiopia; 2 Department of Psychiatry Nursing, Wollega University, Nekemte, Oromia Ethiopia; 3 Department of Paediatric Nursing, Wollega University, Nekemte, Oromia Ethiopia; 4 Department of Comprehensive Nursing, Wollega University, Nekemte, Oromia Ethiopia

**Keywords:** Attention Deficit Hyperactivity Disorder, Misconception, Western Ethiopia

## Abstract

**Objective:**

Teachers’ misconception on Attention Deficit/Hyperactivity Disorder (ADHD) in general and the implementation of effective educational strategies for children with this problem in particular is one obstacle that largely impacts the academic and overall success of school children with this problem. In Ethiopia, despite there are thousands of school children with this ADHD, no studies have been conducted to examine school teachers’ understanding about problem. This research was therefore aimed to investigate primary school teachers’ misconceptions about ADHD in Western Ethiopia.

**Result:**

In this study, 76.2% of respondents had misconception on general awareness of ADHD. More than half (62.7%) of them had misconceptions on the diagnosis and on 81% had misconceptions regarding treatment of the problem. Concerning teachers’ misconception on the contemporarily recommended educational placement of students with ADHD, 141 (68.3%) have said that such students should be placed in part time special education. The findings of this research have clearly indicated that primary school teachers have a wide range of misconceptions about the ADHD. It also reflects the need of equipping teachers with basic knowledge of ADHD which also enables them provide effective support for students with this exceptionality.

## Introduction

Attention Deficit/Hyperactivity Disorder is one of the frequently diagnosed psychiatric problems in childhood. The American Psychiatric Association estimated the prevalence rate of ADHD to be 3–7% among school-age children [1]. Also, American Academy of Pediatrics has been reported that about 9% of school pupils have ADHD [2]. Children with this problem are generally lagged behind in academic performance. As a result, they require superfluous time and power of their teachers to catch up with their class mates [3].

Familiarity of Attention Deficit/Hyperactivity Disorder can amplify teachers’ self-belief in teaching and supervising children with ADHD [4, 5]. Hence, teachers should have required knowledge that enables them to effectively involve in the process of handling these group of children. Regrettably, although very few studies that have been done to assess teacher’s understanding of ADHD in many countries, many teachers, among the studies are performed have a propensity and generous misperceptions about the nature, course, cause, and outcomes of ADHD [6–12].

Teachers who have good understanding about ADHD are most often better prepared to be in a position to offer adequate teaching assistance and render required support for children with ADHD. Moreover, teachers’ misconception on the implementation of effective educational strategies such as appropriate educational placement of such students is one obstacle that largely impacts the academic and overall success of this special population. It was also found that teachers have acknowledged that they have probably no or little opportunity of additional training related to ADHD. As a result, almost all teachers across many countries are distressingly unnamable at least to recognize a child with ADHD and poorly involved in its diagnosis and treatment process [7–16, 26].

In Ethiopia, despite thousands of school children suffer this problem, only a small number of studies have been conducted to examine school teachers’ misconception about this health problem. This research was therefore aimed to investigate primary school teachers’ misconceptions about ADHD in Western Ethiopia.

## Main text

### Study setting

This study was undertaken in 10 government and 20 private primary schools in located in Nekemte town. Nekemte, the capital city of East Wollega zone is found 331 km away from the capital Addis Ababa to the West.

### Method

School based descriptive cross-sectional study design was undertaken. All primary school teachers in the selected schools and those who fulfilled the inclusion criteria were involved. Non probablity conviniance sampling technique was used to recruit the study participants. The the study was undertaken from December 2016 to January 2017.

#### Data collection tool and procedure

The tool used to undertake this research was adopted from previously conducted a research which is also standard questionnaire known as Attention Deficit Disorders Scale (KADDS) [8].

#### Data processing and analysis

After data collection, each questionnaire was checked for completeness before data entry. Data was analysed using SPSS version 20. Frequency tables, graphs and descriptive summaries were used to present the key findings of the study. Binary logistic regression was done to see the existing associations between dependent and independent variables.

### Results

#### Socio-demographic of the study participants

In this study, 234 of total respondents were selected and responded to the provided questionnaires. As about 28 of the distributed questionnaires were incomplete, questionnaires from 206 participants were analyzed making the response rate of 88.03%.

Regarding the key socio demographic characteristics of the study participants, about half (50.5%) were female. Concerning their age, about 62.6% of them were above 40 years old. On the other hand, around 42% of the study participants had more than 16 year of teaching experience. As to their maximum completed level of education, more than half (60.7%) of the selected teachers were first degree holders.

#### Respondents level of misconceptions about ADHD

In this study, respondents’ misconception of ADHD in terms of general awareness, diagnosis, and treatment of the disorder. As revealed in this research, the vast majority (76.2%) of teachers who participated in the study were found to have misconception about Attention Deficit Hyperactivity Disorder.

**Fig. 1:**
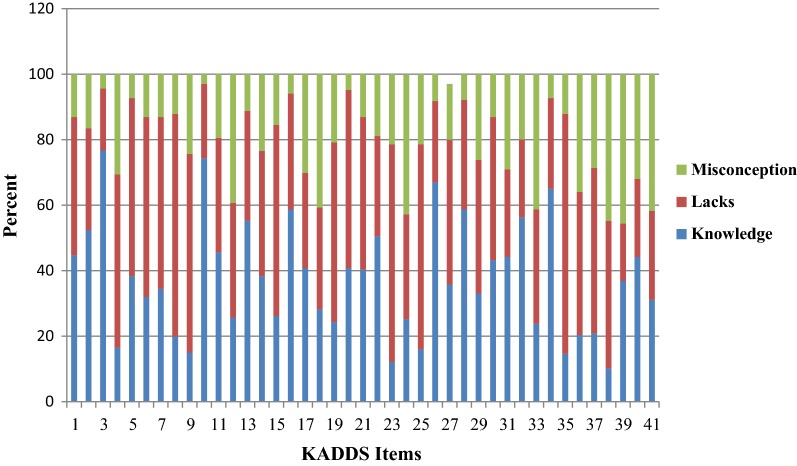
Histogram shows the percentage of respondents’ knowledge, lacks and misconceptions regarding ADHD, Nekemte, 2017

Regarding questions asked to assess how to diagnose a child with ADHD, more than half (55.3%) of the teachers have responded correctly. While around 11.2% of the study participants had misconceptions on the diagnosis of the problem, significant proportion (33.5%) of them did not respond to the provided questions (lacks). Similarly, out of all respondents, 81% of them had misconceptions on treatment of a child with ADHD (Fig. 1).

Teachers’ misconception as to appropriate educational placement for students with ADHD was also assessed. Accordingly, while 141 (68.3%) teachers said the students should be placed into part time special education, 22 (10.7%) and 35 (17.2%), respectively recommend full time general education and full time special education. The remaining 8 (3.8%) of the study participants said that they had not known the current recommendation.

Binary logistic analysis was done between the teachers’ sources of information and separately having general awareness of ADHD as well as with how to diagnose the problem and statistical significance was considered at P < 0.05. Similarly, logistic analysis was done between sources of information on ADHD and misconceptions related to the most appropriate educational placement (Tables 1 and 2).

**Table 1:** Association of respondents’ general awareness on ADHD with their sources of information, Nekemte, Ethiopia, 2017

Source of information	General awareness	Frequency (percentage)	COR	Confidential interval (95%)	p-value	AOR	95% CI	p-value
In-service	No	189 (91.7)	1.69	0.62–4.60	0.30	0.69	0.17–2.67	0.59
	Yes	17 (8.3)	I (R)	I (R)	I (R)	I (R)	I (R)	I (R)
Read any books	No	168 (81.5)	1.61	0.79–3.31	0.18	0.75	0.21–2.60	0.65
	Yes	38 (18.5)	I (R)	I (R)	I (R)	I (R)	I (R)	I (R)
Read any articles	No	180 (87.3)	1.50	0.64–3.51	0.34	0.55	0.14–2.21	0.40
	Yes	26 (12.7)	1 (R)	1 (R)	1 (R)	1 (R)	1 (R)	I (R)
Read any pamphlets	No	178 (86.4)	1.43	0.64–3.23	0.37	0.99	0.25–3.90	0.99
	Yes	28 (13.6)	1 (R)	1 (R)	1 (R)	1 (R)	1 (R)	1 (R)
Watched television	No	153 (74.3)	0.91	0.47–1.76	0.79	1.25	0.54–2.87	0.59
	Yes	53 (25.7)	1 (R)	1 (R)	1 (R)	1 (R)	1 (R)	1 (R)
Searched the internet	No	196 (95.1)	0.19	0.02–1.54	0.12	10.2	1.03–100.9	0.04*
	Yes	10 (4.9)	1 (R)	1 (R)	1 (R)	1 (R)	1 (R)	1 (R)

*Statistically significant

**Table 2:** Association of respondents’ misconception on of ADHD diagnosis with sources of ADHD information, Nekemte, Ethiopia, 2017

Source of information	Diagnosis of ADHD	Frequency and percentage	OR	Confidential interval (95%)	p-value	AOR	95%CI	p-value
In-service	No	189 (91.7)	0.64	0.239–1.75	0.39	1.28	0.317–5.16	0.72
	Yes	17 (8.3)	I (R)	I (R)	I (R)	I (R)	I (R)	I (R)
Read any books	No	168 (81.5)	0.40	0.19–0.82	0.01	0.39	0.11–1.38	0.14
	Yes	38 (18.5)	I (R)	I (R)	I (R)	I (R)	I (R)	I (R)
Read any articles	No	180 (87.3)	0.50	0.21–1.17	0.11	0.66	0.16–2.72	0.57
	Yes	26 (12.7)	1 (R)	1 (R)	1 (R)	1 (R)	1 (R)	I (R)
Read any pamphlets	No	178 (86.4)	0.46	0.207–1.03	0.06	0.83	0.20–3.43	0.80
	Yes	28 (13.6)	1 (R)	1 (R)	1 (R)	1 (R)	1 (R)	1 (R)
Watched television	No	153 (74.3)	1.09	0.57–2.09	0.78	1.98	0.82–4.78	0.12
	Yes	53 (25.7)	1 (R)	1 (R)	1 (R)	1 (R)	1 (R)	1 (R)
Searched the internet	No	196 (95.1)	2.47	0.513–11.98	0.25	6.00	0.86–41.4	0.06
	Yes	10 (4.9)	1 (R)	1 (R)	1 (R)	1 (R)	1 (R)	1 (R)

## Discussion

Teachers play a major role both before and after the child was diagnosed with Attention Deficit/Hyperactivity Disorder (ADHD). During pre-diagnostic period, teachers are the closest and familiar person, apart from family members, who can report crucial information about a child’s in-class behavior and refer him to be evaluated and diagnosed. Following the diagnosis, teachers are expected to help in managing ADHD pupils by adjusting classroom environment and delivering cognitive-behavioral therapy to promote self-control [17]. The results of this study are discussed in the context of the research questions posed earlier elsewhere. In order to examine teachers’ misperception about ADHD within each of the KADDS subscales their responses were grouped to represent the three subscales of KADDS. In this study respondents misconceptions in relation to their general awareness, how to diagnosis, and treatment of ADHD were assessed.

Generally, out of all respondents, 76.2% were found to have misconception on general awareness of ADHD. This is in line with findings study done in Egypt which found that 76.1% respondents in similar study had misconceptions on basic awareness of the problem [18]. Similarly, more than half (62.7%) of this study’s participants have misconceptions on the diagnosis of ADHD. More specifically, the highest proportion of correct response (55.3%, n = 114) was on item 13 that states “It is possible to diagnose in adult ADHD”. Accordingly, significant proportion of the teachers knew that diagnosis of adult with ADHD is easy this leads to their belief to identify the children’s with ADHD in schools with proper training and knowledge. On the other hand, the highest amount of incorrect response (83.5%, n = 172) was on item 4 which says “Children with ADHD are typically more compliant with their fathers than with their mothers”. In this regard this study indicated that majority of the teachers have misconceptions about the change of ADHD symptoms to students. These findings are also proved to be in line with various studies conducted across the world [19, 20].

The third wing in assessing the misconception and/or knowledge level of the school teachers in this study was on treatment of ADHD. With this regard, responses on treatment of ADHD revealed that 81% of the teachers have a misconception. Only 19% were aware that there is no evidence of the effectiveness of the mentioned type of treatment for children with ADHD. Regarding this the highest proportion of incorrect responses (89.8%, n = 185) was on item 39: “Children with ADHD generally display an inflexible adherence to specific routines or rituals”. Less than 10% of the teachers in our survey thought mistakenly that children with ADHD have an inflexible adherence but special education given to these children’s change their obedience. These particular findings are consistent with the findings of previous studies done by Canu and Mancil, Ghanizadeh et al. and few more authors of similar studies [21–24].

Teachers who are knowledgeable about ADHD are better prepared to be in a position to offer adequate teaching assistance and support children with ADHD [25–27]. Teachers’ misconception on appropriate educational placement of students with ADHD is one barrier that affects the implementation of effective strategies for such students. A great deal of literatures indicates that classroom community is a vital part of facilitating a safe and supportive learning environment for students with ADHD. It is particularly crucial that teachers know how to implement different strategies for building an inclusive classroom community to include students with characteristics of ADHD [28].

Previous researches indicated that there is a predominantly negative attitude in children towards their peers and that impairment in peer relations known to be common in children with ADHD. Therefore, the contemporary advice is that it is extremely important to build a classroom community where all students and students with characteristics of ADHD specifically, feel safe and included [29]. Concerning this our study has assessed teachers’ misconception the contemporarily recommended appropriate educational placement for students with ADHD. Majority, 141 (68.3%) of the teachers who have participated on this study said that such students should be placed into part time special education, Significant percentage (17.2%) of them recommended full time special education.

In general, the results of this research clearly indicated that the teachers who participated in this study had a wide range of misconceptions about the ADHD. The results from this study also brought light to the need that teachers themselves should be educated and supported in regarding ADHD through in-service training which ultimately enables the teachers implement effective support for school children with this exceptionality.

## Limitations of the study

As this study was done only on primary school level the research did not know the possible some other misconceptions of teachers at secondary school level. Also, the study did not consider teachers’ misconception of ADHD in rural schools which could be the worst due to variation in information access.

## Data Availability

The raw data supporting our findings are available from authors on a reasonable request.

## Abbreviations

ADHD: Attention Deficit/Hyperactivity Disorder
AOR: adjusted odds ratio
KADDS: Knowledge of Attention Deficit Disorders Scale
